# Relationship between White Matter Hyperintensities categorized by distance from the ventricular surface and cognitive function in Alzheimer`s disease

**DOI:** 10.1002/alz70856_101835

**Published:** 2025-12-25

**Authors:** Hyun‐ju Yang, Jae Min Song

**Affiliations:** ^1^ Jeju national University hospital School of medicine, Jeju‐si, Korea, Republic of (South); ^2^ Jeju Medical Center, Jeju‐si, 63243, Korea, Republic of (South)

## Abstract

**Background:**

White matter hyperintensities (WMH) are common among the elderly. Although WMH contribute to AD pathology, their clinical implications are not fully understood. This study aimed to assess whether WMH predict AD and investigate the association between WMH classified according to the distance from the ventricular surface and cognitive function in AD.

**Method:**

This study enrolled 171 patients with AD and 112 subjects with normal cognitive function. All participants underwent clinical evaluations including brain MRI study, neuropsychological tests using the CEARD‐K neuropsychological assessment battery, and a genetic test. In addition, the Clinical Dementia Rating scale, Charlson comorbidity index, the Korean version of the Geriatric Depression Scale short from were administered. WMH volume was calculated using automated quantification method with SPM and MATLAB image processing software. WMH were classified according to the distance from the ventricular surface. WMH volume data were right‐skewed. Consequently, WMH volume data were logarithmic transformed.

**Results:**

The AD group had a significantly higher mean WMH volume (20.7±18.2 ml) than the NC group (6.8±8.1 ml; *p* <0.001). Logistic regression analysis showed that the log total WMH volume was associated with AD (odds ration = 5.967, 95% CI=1.550‐22.986). Log PVWMH (odds ratio=4.021, 95% CI=1.592‐10.156) and log DWMH volume (odds ratio=2.873, 95% CI=1.227‐6.731) were also linked to AD. Multivariate linear regression analysis showed that total WMH volume in AD was associated with poor performance in categorical verbal fluency test (*p* = 0.008) and word list memory test (*p* = 0.023). JVWMH volume in AD was associated with poor performance on categorical verbal fluency test (*p* = 0.013) and digit span test forward (*p* = 0.037). PVWMH volume in AD was associated with poor performance on categorical verbal fluency test (*p* = 0.011) and word list memory test (*p* = 0.021), whereas DWMH volume showed no association with cognitive dysfunction in AD.

**Conclusion:**

Total WMH, JVWMH and PVWMH were associated specifically with executive function, immediate memory, and working memory, independently of hippocampal atrophy in AD. WMH were associated with AD risk and cognitive dysfunction differentially according to the distance from the ventricular surface.

